# *Gh_FBL43* regulates the resistance of *Gossypium hirsutum* to Verticillium wilt through jasmonic acid and flavonoid-related pathways

**DOI:** 10.3389/fpls.2025.1620947

**Published:** 2025-07-17

**Authors:** Jieyin Zhao, Xuening Su, Wenju Gao, Tingwei Wang, Yuxiang Wang, Quanjia Chen, Yanying Qu

**Affiliations:** Xinjiang Key Laboratory of Crop Biological Breeding, Engineering Research Centre of Cotton, Ministry of Education, College of Agriculture, Xinjiang Agricultural University, Urumqi, China

**Keywords:** Gossypium hirsutum, Verticillium wilt, Gh_FBL43, RNA-Seq, jasmonic acid

## Abstract

F-box-LRR (FBL) genes play crucial roles in the response of plants to pathogen stress. This study involved a systematic analysis of the evolution of the FBL gene family in *Gossypium hirsutum* from a whole-genome perspective, and through expression pattern analysis combined with virus-induced gene silencing (VIGS), *Gh_FBL43* was identified as a gene associated with resistance to Verticillium wilt in *G. hirsutum*. Further RNA-seq analysis revealed key pathways and genes regulated by *Gh_FBL43*. The *G. hirsutum* genome contains 57 FBL genes, which can be divided into five subgroups that were relatively conserved during the evolution of cotton. Expression analysis revealed that the expression level of *Gh_FBL43* significantly increased under Verticillium wilt stress, with notable differences observed among extreme varieties. VIGS-meditated silencing of *Gh_FBL43* was performed, and the leaves of the silenced plants presented chlorosis and necrosis, with the disease severity index (DSI) and disease severity rate (DSR) being significantly greater than those of the empty vector control plants. RNA-seq data from *Gh_FBL43-*silenced and control plants at 0 h and 24 h post-infection revealed 10,928 differentially expressed genes (DEGs), including 2,051 shared DEGs. Enrichment analysis combined with expression pattern analysis indicated that the silencing of *Gh_FBL43* reduced the expression of genes in jasmonic acid (JA) and flavonoid-related pathways. In conclusion, our findings demonstrate the important role of the *Gh_FBL43* gene family in conferring resistance to Verticillium wilt in *G. hirsutum*, potentially regulating this resistance through JA and flavonoid-related pathways, thereby laying a foundation for further elucidation of the molecular mechanisms by which *Gh_FBL43* confers resistance to Verticillium wilt.

## Introduction

Cotton is not only an important raw material for the textile industry but also a significant source of feed and biofuels (Saleem et al., 2024). *Gossypium hirsutum* plays a crucial role in cotton production; however, Verticillium wilt, a disease caused by the pathogen *Verticillium dahliae*, severely restricts the yield and quality of *G. hirsutum* (Zhu et al., 2023). Each year, the average yield loss due to cotton wilt is approximately 10–35%. Verticillium wilt is the most severe vascular disease affecting cotton, and the primary causative pathogen is *V*. *dahliae*, a semibiotrophic filamentous fungus that mainly infects plant roots through the soil (Kowalska, 2021). The mycelia penetrate the root surface and reside in the vascular bundles, leading to plant death. The main pathogenic mechanism involves the obstruction of xylem vessels and the production of toxins (Zhang et al., 2022). Increased transpiration and respiration in the aerial parts of the cotton plant lead to an imbalance of moisture within the plant, resulting in symptoms such as leaf wilting, yellowing, and browning of vascular bundles, ultimately leading to plant death (Bhandari et al., 2020).

Plants experience attacks from various pathogens during their growth and development. To resist such attacks, plants have developed multiple defense mechanisms, one of which is controlled by resistance (R) genes (Kourelis and Van Der Hoorn, 2018). R genes are capable of directly conferring disease resistance and can specifically recognize and activate plant defense responses and systemic acquired resistance (Kiraly et al., 2007). In recent years, several R genes have been cloned for research on plant disease resistance. Systematic analysis of R genes has revealed that they have similar structures and that most possess conserved domains (TIR-NBS-LRR) (Zhou et al., 2022). Since F-box-LRR (FBL) genes contains R gene structural domains, they play a crucial role in the process of plant disease resistance (Dou et al., 2025). The F-box protein ACRE189/ACIF1 can regulate the activation of programmed cell death and defense responses in tobacco and tomato in response to pathogens (van den Burg et al., 2008). In rice, overexpression of the F-box protein-encoding gene *OsDRF1* enhances disease resistance (Cao et al., 2008). In maize, overexpression of *ZmFBL41* leads to decreased resistance to gray leaf spot disease, whereas knockout of *ZmFBL41* increases resistance (Li et al., 2019). Further experimentation has shown that *ZmFBL41* can interact with *ZmCAD*, regulating resistance to gray leaf spot disease by modulating the accumulation of lignin. Increasing evidence indicates the significant role of F-box proteins in plant disease resistance (Yang et al., 2024). Many hormone receptors contain F-box structural domains, such as the *Arabidopsis thaliana MAX2* protein, which is an F-box protein with an LRR domain at the C-terminus. *AtMAX2* blocks bacterial invasion by closing stomata (Zheng et al., 2023). Silencing the LRR gene *GbRVd* in cotton through virus-induced gene silencing (VIGS) significantly decreased the levels of salicylic acid (SA), nitric oxide (NO) and hydrogen peroxide, resulting in decreased resistance to wilt disease (Yang et al., 2016).

RNA-seq has high sequencing throughput, a wide range of applications, and high sensitivity, offering significant convenience for screening differentially expressed genes (DEGs) and investigating key DEGs (Ren et al., 2024). Through RNA-seq, many key regulatory networks and pathways related to plant growth, development, and stress resistance have been revealed, providing important clues and insights for elucidating the molecular mechanisms associated with important genes and traits (Ebeed and Ceasar, 2024). Transcriptomic analysis of transgenic *A. thaliana* overexpressing *ScDREB10* suggested that *ScDREB10* may regulate plant tolerance to abiotic stress by affecting phenylpropanoid biosynthesis and starch and sucrose metabolism (Liang et al., 2024). Transcriptomic sequencing of transgenic peanut lines overexpressing MYB revealed that the MAPK cascade, ethylene, auxin, and abscisic acid-related genes are crucial for the response to abiotic stress (Raziq et al., 2024). An analysis of the transcriptome and metabolome of *A. thaliana* lines transformed with the rice *Os-LBD37*/*ASL39* gene revealed significant changes in nitrogen-containing metabolites in the transgenic lines, leading to the identification of *Os-LBD37*/*ASL39* as a nitrogen metabolism regulatory gene in rice (Kusano and Saito, 2012). These results indicate that transcriptomics has significant potential for revealing the regulatory mechanisms of important functional genes.

By studying the evolution of gene families, sufficient information can be gained regarding their diversity and biological functions (Goyal et al., 2023). In recent years, with advancements in sequencing technologies and a reduction in sequencing costs, the sequences of plant genomes have been continuously improved and updated, laying the foundation for the identification and study of gene family evolution and function at the whole-genome level (Cheng et al., 2021). Although candidate genes related to Verticillium wilt have been identified in cotton, analyses of the regulatory mechanisms are severely lacking (Zhao et al., 2021; Cui et al., 2021; Ayyaz et al., 2025). The FBL family of genes is potentially involved in plant disease resistance. However, the evolution, function, and classification of this gene family in *G. hirsutum* have not been systematically studied. This study analyzes the evolutionary relationships, gene structures, and promoter cis-regulatory elements of *G. hirsutum* FBL genes. Through expression pattern analysis combined with VIGS, it was determined that *Gh_FBL43* plays a significant role in the resistance of *G. hirsutum* to Verticillium wilt. The RNA-seq data before and after infection of the silenced and control plants revealed the regulatory pathways through which *Gh_FBL43* confers resistance to Verticillium wilt. These results will further broaden our understanding of the role of FBL genes in plants and provide valuable insights for deeper analysis of the regulatory mechanisms of *Gh_FBL43* in combating Verticillium wilt.

## Materials and methods

### Plant material

The wilt-resistant variety Zhongzhimian 2 and the wilt-susceptible variety Junmian 1 were selected for expression analysis under wilt disease stress. The materials were provided by the Cotton Innovation Team of the College of Agriculture at Xinjiang Agricultural University and were planted in the Cotton Cultivation Laboratory of the Key Laboratory of Agriculture and Biotechnology at Xinjiang Agricultural University. Seeds of uniform fullness were selected and sown in a mixture of perlite and sterilized soil (ratio of 1:2), with three seeds per pot. The laboratory was maintained at a daytime temperature of 25.0 to 28.0°C and illuminated with incandescent lighting. Germination occurred under a cycle of 16 hours of light and 8 hours of darkness, with a relative humidity of 80%. When the cotton plants developed three true leaves, the bottom-tear root method was used to inoculate the plants with the wilt pathogen V991, with three replicates for each treatment. Root tissue samples were collected at 0, 1, 3, 6, 12, 24 and 48 h post-wilt stress. All the samples were quickly frozen in liquid nitrogen and stored at -80°C.

### Identification of cotton FBL gene family members and analysis of their physiochemical properties

The genomic data of *G. hirsutum*, *G. barbadense*, *G. arboreum* and *G. raimondii* were downloaded from the CottonGen database (https://www.cottongen.org/). The hidden Markov model of the F-box domain (PF00646) and HMMER 3.2.1 software (http://hmmer.org/) were used to identify the F-box protein sequences in *G. hirsutum*, *G. barbadense*, *G. arboreum* and *G. raimondii* (Prakash et al., 2017). We verified whether the candidate sequences possessed complete F-box and LRR domains using the CDD database (https://www.ncbi.nlm.nih.gov/Structure/cdd/wrpsb.cgi), ultimately obtaining the FBL protein sequences for *G. hirsutum*, *G. barbadense*, *G. arboreum* and *G. raimondii*. The physicochemical properties of *G. hirsutum* FBL proteins were predicted via the online tool ExPASy (https://web.expasy.org/protparam/).

### Phylogenetic analysis of the FBL gene family in *G. hirsutum*

With the default parameters of ClustalW in MEGA X software, multiple sequence alignments of FBL protein sequences from *G. hirsutum*, *G. barbadense*, *G. arboreum* and *G. raimondii* were performed (Kumar et al., 2018). The phylogenetic tree was constructed using the neighbor-joining (NJ) method with a bootstrap value of 1000. The online tool EvolView (https://www.evolgenius.info/evolview/) was employed to enhance the visualization of the phylogenetic tree. The MEME online tool (http://alternate.meme-suite.org/tools/meme) was used to analyze the motifs of the *G. hirsutum* FBL gene family, with the number of functional domains set to 10. The xml file generated by MEME, the nwk file of the phylogenetic tree, and the GFF3 file of gene structures were visualized using TBtools software (Chen et al., 2023).

### Analysis of cis-acting elements and expression patterns of *G. hirsutum* FBL genes

The 2000 bp sequence upstream of the start codon of the FBL gene from *G. hirsutum* was extracted and submitted to the PlantCARE (http://bioinformatics.psb.ugent.be/webtools/plantcare/html/) online tool to predict the cis-regulatory elements in the gene promoter, and TBtools software was used for visualization (Chen et al., 2023). The transcriptome sequencing data of *G. hirsutum* were downloaded from the NCBI database (accession numbers: PRJNA248163 and PRJNA532694). Fastp (version 0.23.4) software was used for filtering and quality control of the raw data (Chen et al., 2018). HISAT2 software was used to align the filtered data with the *G. hirsutum* TM-1 reference genome (https://www.cottongen.org/species/Gossypium_hirsutum/ZJU-AD1_v2.1), and StringTie software was used for expression quantification (Kim et al., 2019). Gene expression levels were determined via the FPKM method, and expression heatmaps were created via TBtools software.

### qRT–PCR analysis

Total RNA extraction was performed using an RNA extraction kit (Tiangen, China). cDNA was synthesized from RNA using the M-MLV RTase cDNA Synthesis Kit (TaKaRa, Japan). The qRT–PCR primers used were designed using Primer Premier 5.0 software (**Supplementary Table S1**), with the *GhUBQ7* gene from *G. hirsutum* used as the internal control. The qRT–PCRs were conducted using the ChamQ Universal SYBR qPCR Master Mix (Novogene Bioinformatics Technology Co., Ltd., Nanjing). The reaction program consisted of 30 seconds at 95°C for initial denaturation, followed by 40 cycles of 10 seconds at 95°C for denaturation, 30 seconds at 60°C for annealing, and 20 seconds at 72°C for extension. Each sample was examined in triplicate. The quantitative data were analyzed using the 2^–ΔΔCT^ method (Livak and Schmittgen, 2001).

### Cotton VIGS

VIGS experiments involving tobacco rattle virus (TRV) were conducted on cotton (Bachan and Dinesh-Kumar, 2012). The vectors pTRV2 and pTRV1 containing specific regions of *Gh_FBL43* were subsequently transformed into Agrobacterium GV3101. The restriction sites selected were BamHI and KpnI, and the primer sequences for amplifying the *Gh_FBL43* fragment are shown in **Supplementary Table S1**. A mixture of Agrobacterium cells containing pTRV1, pTRV2, or the derived plasmids was transformed into 7-day-old seedlings. After inoculation, the seedlings were washed with deionized water to remove excess Agrobacterium residue and were grown at 25°C under a 16 hour/8 hour light/dark cycle. After 2 weeks of cultivation, the plants were inoculated with the yellow wilt pathogen strain V991. At least 30 seedlings were treated in each trial, and the experiments were repeated three times to evaluate plant disease severity at 21 days post-inoculation.

### RNA-seq: sequencing and analysis

Additionally, RNA-seq with three biological replicates was conducted on samples from the control group with empty vectors and the *Gh_FBL43*-silenced plants at 0 h and 24 h post-inoculation. The samples were transported to Aide Sen Biotechnology Co., Ltd., in Xinjiang (Urumqi, China) for sequencing using dry ice. RNA was extracted via a polysaccharide and polyphenol plant total RNA extraction kit, and the extraction process was carried out according to the instructions. After RNA extraction, a Nanodrop was used to determine the RNA purity (OD260/280) and concentration and to determine whether the nucleic acid absorption peak was normal. The integrity of the RNA was accurately detected with an Agilent 2100 instrument. The detection indicators included the following: the RIN value, 28S/18S ratio, whether the chromatogram baseline was increased, and the 5S peak. After the concentration and purity of the extracted RNA samples were determined, a library was constructed using an initial total amount of 1 μg for each sample. Preliminary quantification was performed using a Qubit 3.0 fluorometer, followed by detection of the inserted fragments in the library using the Qsep400 high-throughput analysis system. The effective concentration of the library was accurately quantified using the Q–PCR method to ensure library quality (effective library concentration > 2 nM). The libraries were sequenced on an Illumina HiSeq 2500 (California, USA) platform to generate 150 bp paired-end reads. Fastp (version 0.23.4) software was used to remove reads containing adapters and low-quality reads, resulting in clean data (Chen et al., 2018). The clean reads were aligned to the *G. hirsutum* TM-1 reference genome (https://www.cottongen.org/species/Gossypium_hirsutum/ZJU-AD1_v2.1) using HISAT2 software, and the aligned reads were quantified with StringTie (Kim et al., 2019). DEGs were screened on the basis of gene count values in various samples using DEseq2 software, applying |log2fold change| ≥ 1 and FDR < 0.01 as selection criteria (Love et al., 2014). The fragments per kilobase of exon model per million mapped fragments (FPKM) for each gene was calculated based on its length, and the expression amount mapped to that gene was calculated. R language PCAtools was used to decompose the expression data (FPKM) of all genes into n principal components to describe the characteristics of the original dataset. For all DEGs, Gene Ontology (GO) and Kyoto Encyclopedia of Genes and Genomes (KEGG) enrichment analyses were conducted via the hypergeometric test method with the R language ClusterProfiler (version 4.14.4) package (Wu et al., 2021).

## Results

### Identification of the FBL gene family in cotton

To study the variation in the copy number of the FBL gene family during the evolution of cotton, a total of 171 FBL family genes were identified in the genomes of *G. hirsutum*, *G. barbadense*, *G. arboreum* and *G. raimondii*. In the two diploid cotton species (*G. arboreum* and *G. raimondii*), the number of FBL family genes is close to half of that in *G. hirsutum*. On the basis of the positions of 57 sequences in the chromosomes of *G. hirsutum*, the FBL genes in this species were named sequentially as *Gh_FBL1* to *Gh_FBL57* (**Supplementary Table S2**). The 57 *G. hirsutum* FBL genes are distributed across 24 chromosomes, with two FBL genes (*Gh_FBL28* and *Gh_FBL34*) located on scaffold chromosomes (**Supplementary Figure S1**). The A subgenome contains 28 FBL genes, whereas the D subgenome contains 29 FBL genes. Except for there being one more FBL gene on chromosome D07 than on chromosome A07, the numbers of A subgenome and D subgenome genes in the remaining chromosomes are completely consistent. Overall, there remains a strong correspondence between the A subgenome and the D subgenome, which is consistent with the evolutionary relationships of cotton.

### Evolutionary analysis of the FBL gene family

**Figure 1:**
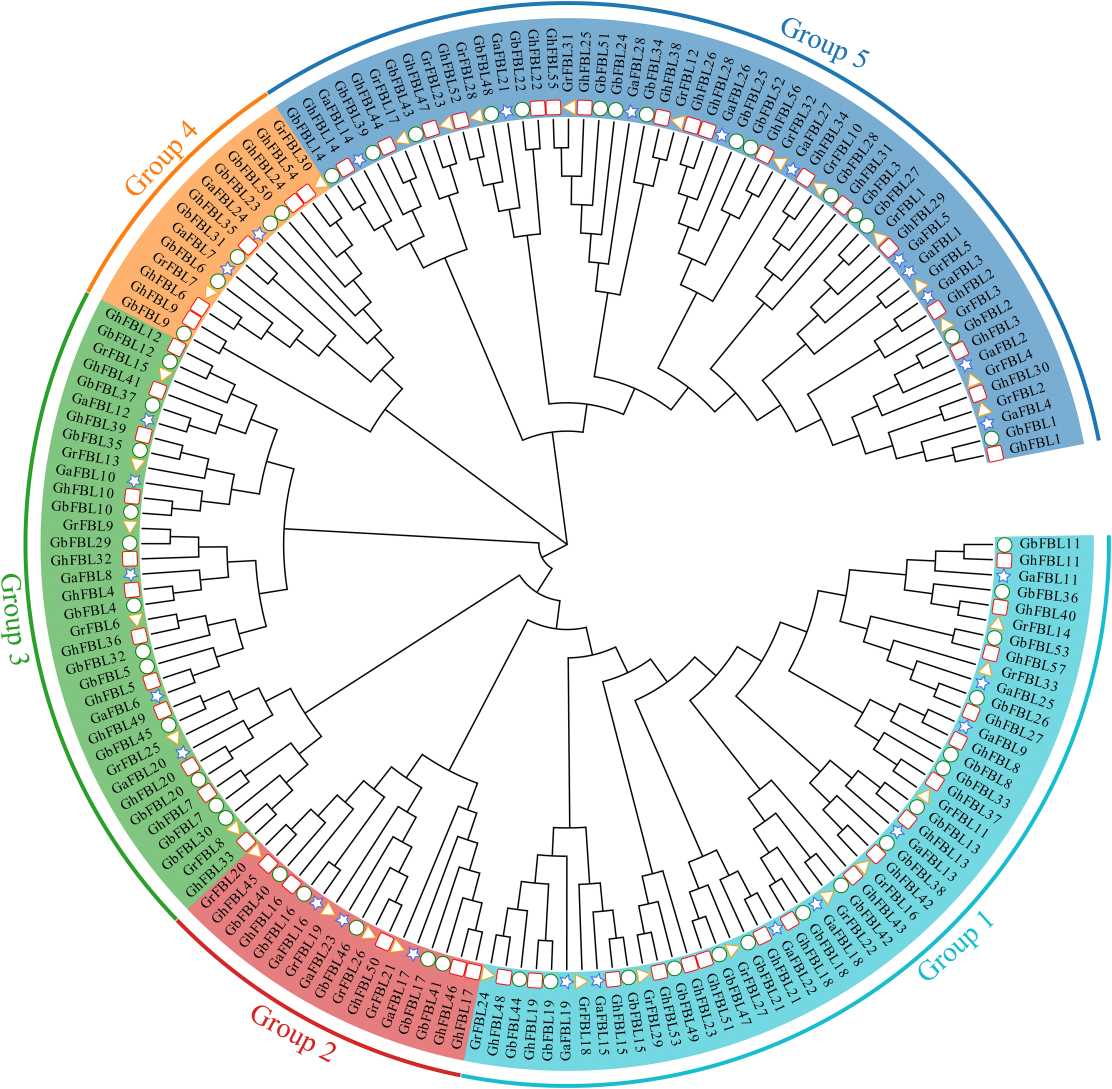
Phylogenetic tree of the cotton FBL gene family, with different colors representing different groups. The pentagon represents the *G. arboreum* FBL genes, the triangle represents the *G. raimondii* FBL genes, the circle denotes the *G. barbadense* FBL genes, and the square indicates the *G. hirsutum* FBL genes.

To better understand the evolutionary relationships of the cotton FBL gene family, a phylogenetic tree was constructed based on 171 FBL protein sequences from *G. hirsutum*, *G. barbadense*, *G. arboreum* and *G. raimondii*. Evolutionary tree analysis indicated that the cotton FBL gene family can be divided into five subgroups (**Figure 1**). Group 1 contains 50 FBL genes, Group 2 contains 17 FBL genes, Group 3 contains 35 FBL genes, Group 4 contains 14 FBL genes, and Group 5 contains 55 FBL genes. The number of FBL genes in each subgroup in both *G. hirsutum* and *G. barbadense* was approximately twice that in *G. arboreum* and *G. raimondii*. This finding is consistent with the earlier analysis results and aligns with the evolutionary relationships among the cotton species, suggesting that the FBL genes are relatively conserved during the evolution of cotton. Although Group 4 has fewer members, they have been conserved throughout the evolution of cotton, indicating that they may play crucial roles in biological processes.

### Evolution, gene structure, and motif analysis of the FBL gene family in *G. hirsutum*

**Figure 2:**
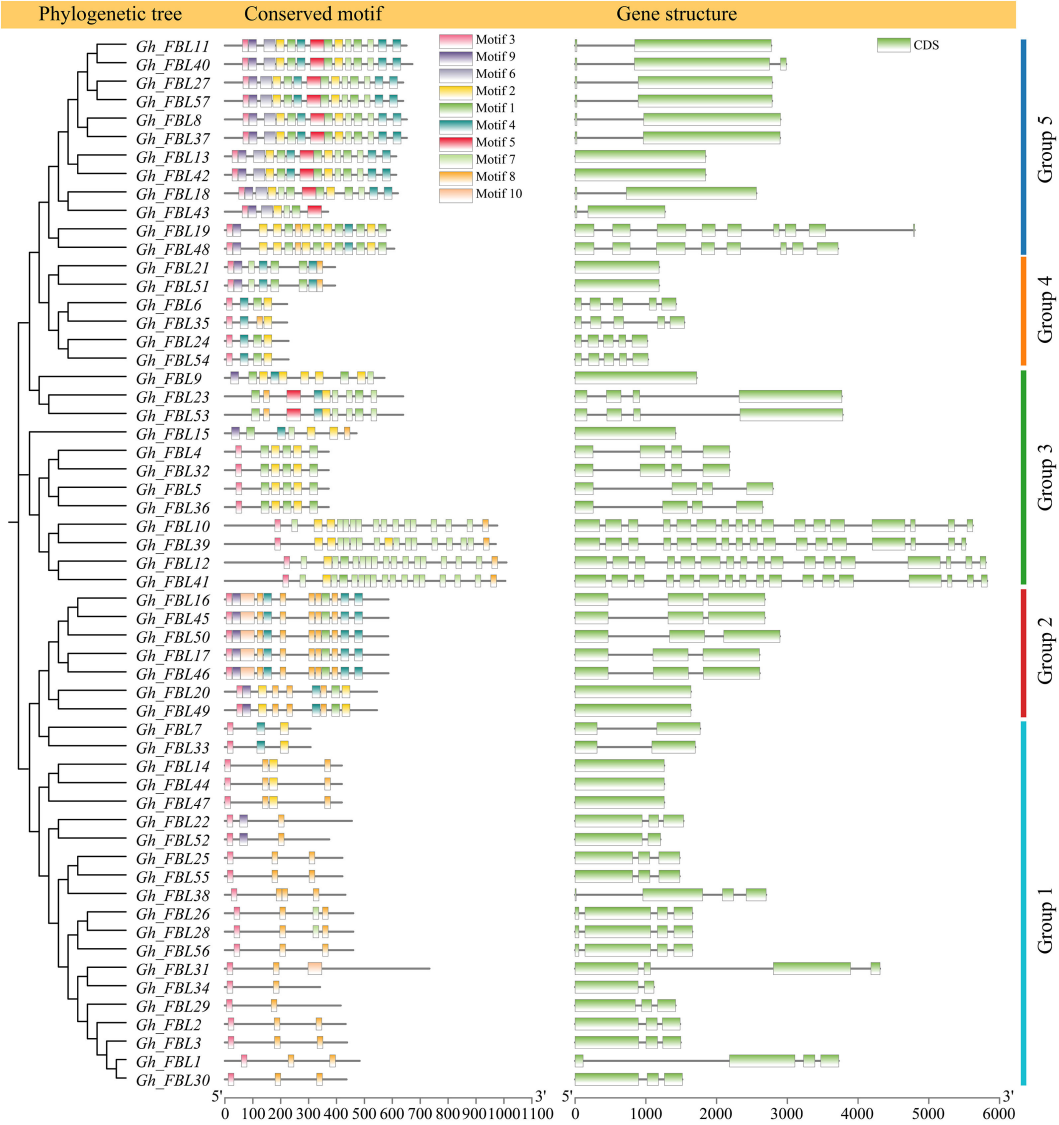
Gene structure and motif analysis of the FBL gene family in *G. hirsutum*.

Analysis of the phylogenetic tree, gene structure, and motifs was conducted on the basis of the full-length sequences, coding sequences (CDSs), and protein sequences of the *G. hirsutum* FBL genes (**Figure 2**). Most genes in Group 1 contain three exons and consist of two motifs (motif 3 and motif 10). In Group 2, genes contain either one or three exons and include seven motifs (motif 1, motif 3, motif 4, motif 7, motif 8, motif 9, and motif 10). Group 3 genes possess four or seventeen exons and comprise five motifs (motif 1, motif 2, motif 3, motif 7, and motif 8). Genes in Group 4 have either one or five exons and consist of four motifs (motif 1, motif 3, motif 4, and motif 8). Most genes in Group 5 contain two exons and encompass eight motifs (motif 1, motif 2, motif 3, motif 4, motif 5, motif 6, motif 7, and motif 9).

### Analysis of promoter homeopathic elements of the *G. hirsutum* FBL gene family

**Figure 3:**
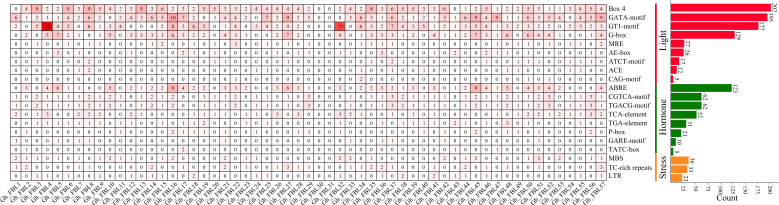
Analysis of homeopathic elements of FBL gene family promoters in G. hirsutum. The numbers in the heatmap represent the number of cis-acting elements of a specific gene, and the bar graph on the right represents the number of cis-acting elements of all FBL genes in *G. hirsutum*.

To investigate the potential functions of the FBL gene in *G. hirsutum*, the promoter region 2000 bp upstream of the GhFBL gene was analyzed, revealing a varying number of stress- and hormone-responsive cis-acting elements (**Figure 3**). A total of three types of cis-acting elements related to the stress response were identified: the MYB-binding site involved in drought inducibility (MBS), the cis-acting element involved in defense and stress responsiveness (TC-rich repeats), and the cis-acting element involved in low-temperature responsiveness (LTR). Additionally, eight hormone-related cis-acting elements were identified, including the cis-acting element involved in abscisic acid responsiveness (ABRE), the cis-acting regulatory elements involved in methyl jasmonate (MeJA) responsiveness (the CGTCA motif and TGACG motif), the cis-acting element involved in salicylic acid responsiveness (the TCA element), the auxin-responsive element (the TGA element), and the gibberellin-responsive elements (the P-box, GARE motif, and TATC box). Among these, the elements related to MeJA and abscisic acid were the most abundant, with 124 and 123 such elements, respectively. Each GhFBL promoter contained a different number and type of cis-acting elements, suggesting that these elements may participate in cotton growth and development and in response to environmental stress through different signaling pathways.

### An analysis of the tissue-specific organization and expression patterns of the FBL gene family in *G. hirsutum* under wilt disease stress

**Figure 4:**
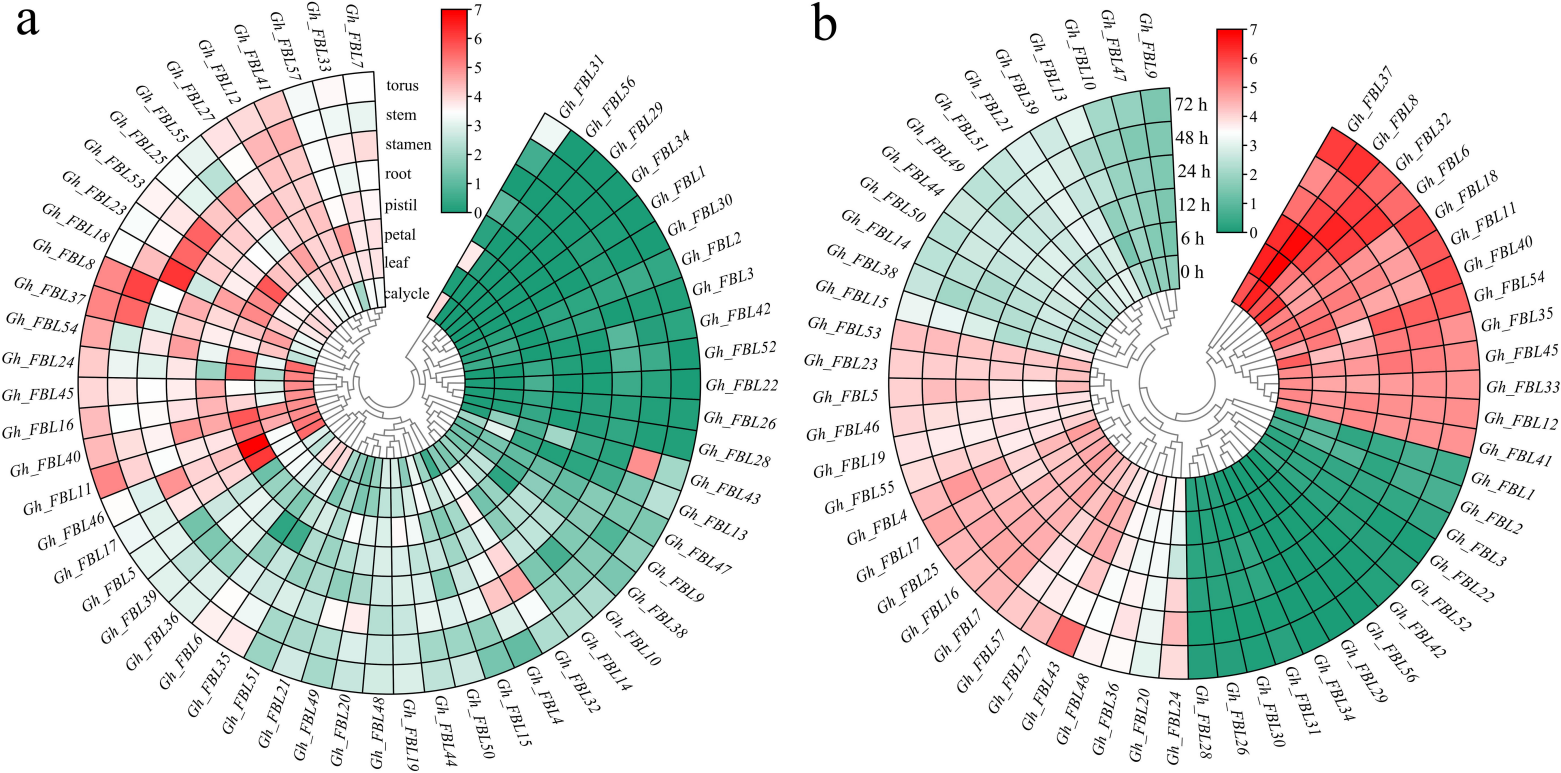
Analysis of the expression patterns of the land cotton FBL gene family. **(a)** Heatmap of tissue-specific expression, log10 (FPKM+1) was used to standardize gene expression. **(b)** Heatmap of expression levels at different time points under Verticillium wilt stress, log10 (FPKM+1) was used to standardize gene expression.qRT–PCR analysis of the FBL gene in cotton under Verticillium wilt stress.

Gene expression patterns are typically related to the functions of genes. The expression patterns of cotton FBL genes in different tissues and at various time points under Verticillium wilt stress were examined via RNA-seq analysis (**Figure 4**). The analysis of tissue-specific expression patterns revealed that 78.95% of the cotton FBL genes presented tissue-specific expression, with the highest expression levels observed primarily in the calyx, petal, stamen, and torus (**Figure 4a**). Following Verticillium wilt occurrence, more than 64.91% of the cotton FBL genes presented changes in expression levels, indicating that FBL family genes may play a significant role in the response of cotton to Verticillium wilt stress (**Figure 4b**). Among them, the five genes (*Gh_FBL20, Gh_FBL24, Gh_FBL36, Gh_FBL43* and *Gh_FBL48*) that presented the most pronounced changes were selected as candidate genes for further validation of cotton resistance to Verticillium wilt.

**Figure 5:**
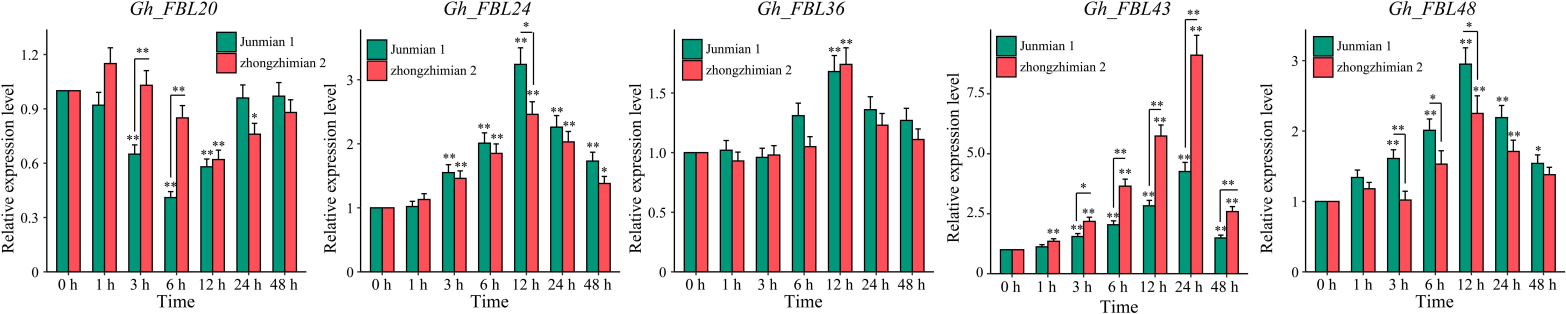
Expression patterns of five candidate genes under yellow wilt disease stress. The results are presented as the means ± SDs (p values were calculated via t-test, n = 3, *p < 0.05, **p < 0.01).

Through RNA-seq, five candidate genes (*Gh_FBL20, Gh_FBL24, Gh_FBL36, Gh_FBL43* and *Gh_FBL48*) involved in the resistance of cotton to Verticillium wilt were ultimately screened. The transcription levels of these five genes were measured under Verticillium wilt stress conditions via qRT–PCR in resistant (Zhongzhimian 2) and susceptible (Junmian 1) extreme varieties (**Figure 5**). Four genes (*Gh_FBL24, Gh_FBL36*, *Gh_FBL43* and *Gh_FBL48*) presented significantly increased expression across different time points in both extreme varieties under Verticillium wilt stress, whereas *Gh_FBL20* presented a significant decrease in expression in expression post-infection. Notably, *Gh_FBL43* expression peaked (fold change > 9) at 24 hours post-infection, and this gene presented significant differences in expression levels between the two extreme varieties at 3 hours post-infection. *Gh_FBL43* is suggested as a candidate gene for further functional analysis of cotton resistance to Verticillium wilt.

### Cotton VIGS

**Figure 6:**
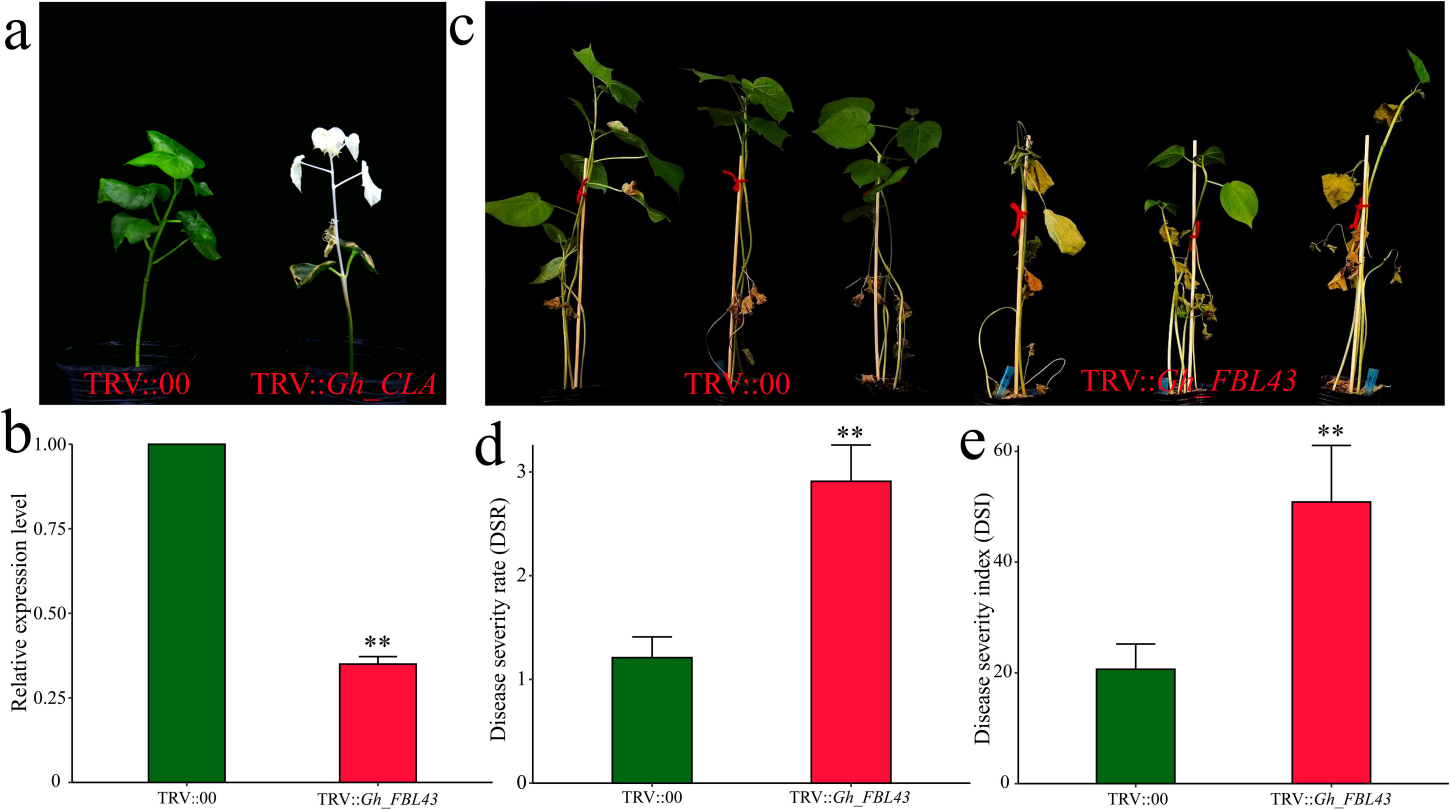
VIGS phenotype and disease severity index of Gh_FBL43 in cotton. **(a)** Chlorosis phenotype observed 14 days after TRV::CLA injection. **(b)** Determination of *Gh_FBL43* silencing efficiency. **(c)** Phenotype 21 days post bacterial wilt infection after silencing *Gh_FBL43*. **(d)** Disease severity after silencing G*h_FBL43*. **(e)** Disease index after silencing *Gh_FBL43*. The error bars represent the mean values of three repeats ± SDs (p values were calculated via a t-test, n = 3, **p < 0.01).

To validate the role of *Gh_FBL43* in cotton resistance to Verticillium wilt, we silenced *Gh_FBL43* in *G. hirsutum* using VIGS. Cotyledons of seven-day-old cotton seedlings were injected with Agrobacterium cells carrying pTRV1 or pTRV2-*GhCLA*. Fifteen days later, the new true leaves presented a distinct chlorosis phenotype, indicating that the VIGS system used in this study successfully silenced the gene (**Figure 6a**). The results of the qRT–PCR analysis of the *Gh_FBL43-*silenced plants compared with the control plants revealed that the expression level of *Gh_FBL43* in the silenced plants was significantly lower than that in the control plants treated with an empty vector (**Figure 6b**). After confirming the silencing of the *Gh_FBL43* gene, we inoculated the silenced and control plants with the V991 strain of the Verticillium wilt fungus using a root injury-based method. Twenty-one days post-inoculation, the leaves of the silenced plants presented significant yellowing and necrosis, whereas those of the control plants remained in good growth conditions with no noticeable loss of greenness (**Figure 6c**). Further phenotypic investigation revealed that the disease severity and disease index of the *Gh_FBL43-*silenced plants were significantly greater than those of the control plants (**Figures 6d, e**).

### Comprehensive analysis of RNA-seq data

**Figure 7:**
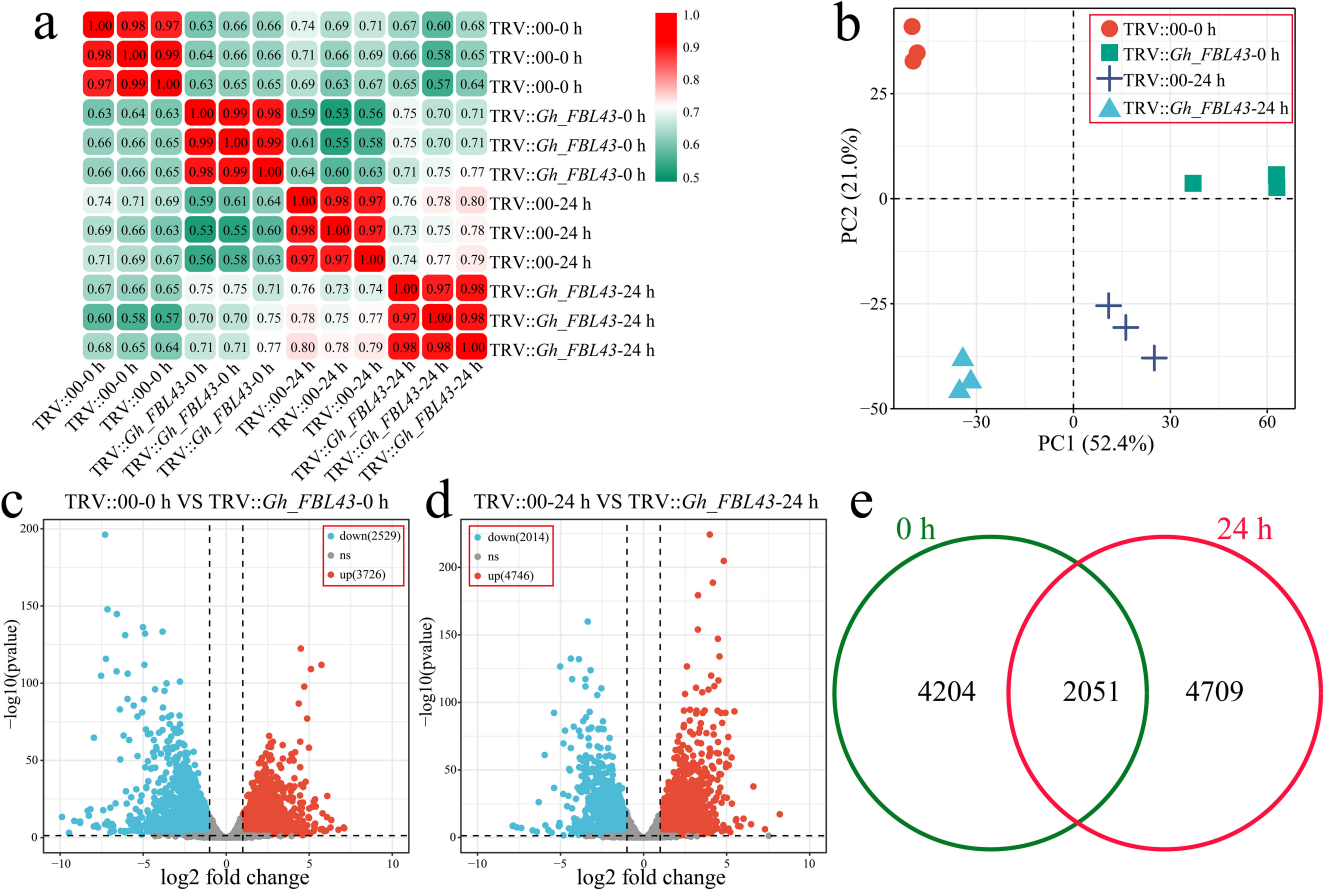
Overall RNA-seq analysis of *Gh_FBL43-*silenced and control cotton plants at 0 h and 24 h post infection with the Verticillium wilt pathogen. **(a)** Correlation analysis of samples. **(b)** PCA of samples. **(c)** Volcano plot of differential expression analysis results at 0 h post infection. **(d)** Volcano plot of differential expression analysis results at 24 h post infection. **(e)** Venn diagram of specific and shared DEGs at different time points post infection.

To clarify the pathway by which *Gh_FBL43* regulates cotton resistance to *V. dahliae*, RNA-seq was conducted on both *Gh_FBL43*-silenced and control cotton plants at 0 hours and 24 hours post-inoculation with the pathogen, with three biological replicates for each sample. A total of 81.52 Gb of clean data was obtained after the raw data were filtered, with each sample yielding more than 6.05 Gb of clean data. The percentage of Q20 bases was above 98.23%, and the percentage of Q30 bases exceeded 94.58%, with the GC content exceeding 44.14% (**Supplementary Table S3**). The Pearson correlation coefficients among the three replicates of the same sample were greater than 0.97 (**Figure 7a**). Principal component analysis (PCA) revealed that samples of the same biological replicate clustered together, indicating high reliability and reproducibility of the transcriptomic data (**Figure 7b**). Through differential expression analysis, a total of 6,255 DEGs were identified between the *Gh_FBL43*-silenced and control plants at 0 hours post-inoculation, of which 3,726 were upregulated and 2,529 were downregulated (**Figure 7c**). At 24 hours post-inoculation, 6,760 DEGs were identified between the two groups, consisting of 4,746 upregulated and 2,014 downregulated genes (**Figure 7d**). Overall, 10,928 DEGs were identified between *Gh_FBL43*-silenced and control plants pre- and post-inoculation, with 4,204 unique DEGs at 0 h and 4,709 unique DEGs at 24 h, including 2,015 shared DEGs (**Figure 7e**).

### Enrichment analysis of DEGs

**Figure 8:**
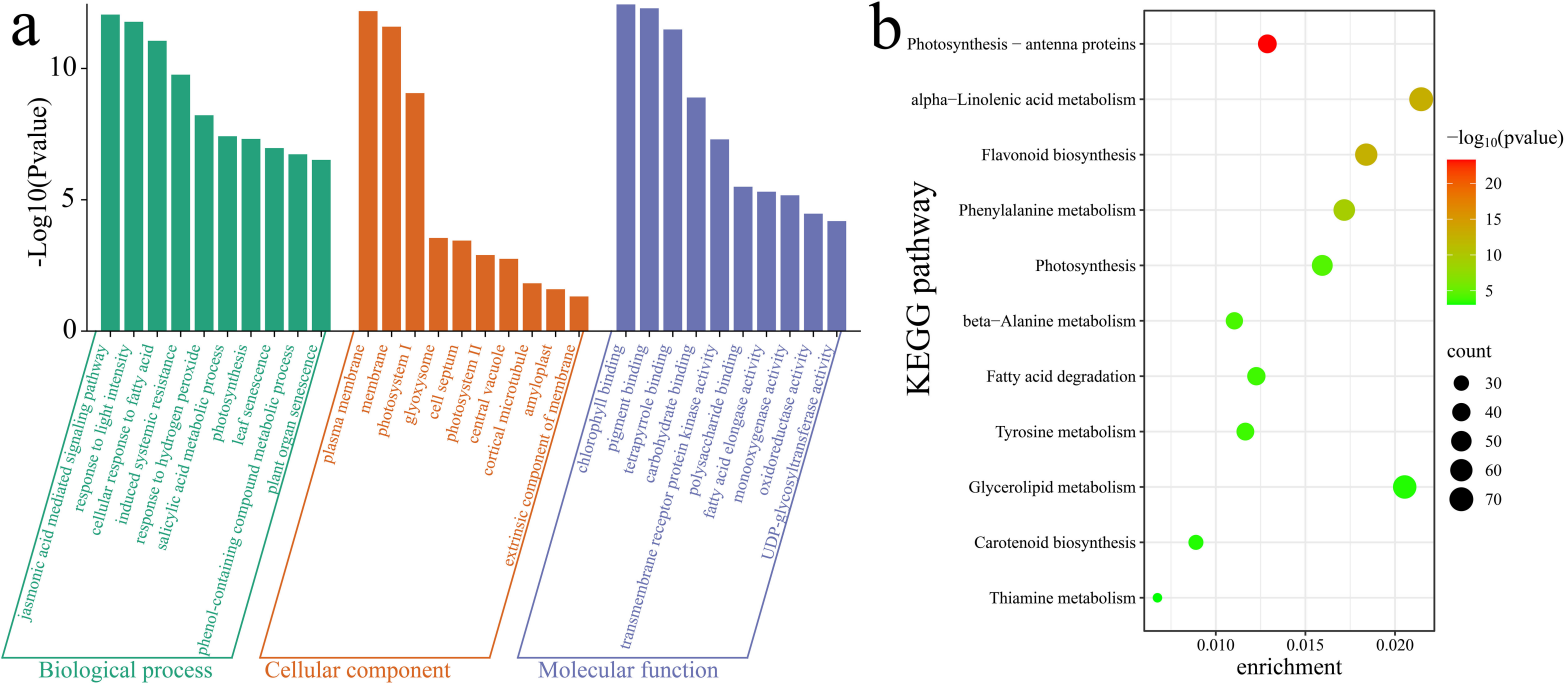
Enrichment analysis of DEGs. **(a)** GO enrichment analysis of DEGs. **(b)** KEGG enrichment analysis of DEGs.

To further elucidate the functions and pathways associated with the 10,928 DEGs, a Gene Ontology (GO) enrichment analysis was first conducted on these DEGs. Notable enrichment was observed in biological process terms such as the jasmonic acid (JA)-mediated signaling pathway, response to light intensity, cellular response to fatty acid, induced systemic resistance, response to hydrogen peroxide, salicylic acid metabolic process, photosynthesis, leaf senescence, phenol-containing compound metabolic process, and plant organ senescence (**Figure 8a**). Additionally, a KEGG enrichment analysis highlighted significant enrichment in the following pathways: photosynthesis - antenna proteins, alpha-linolenic acid metabolism, flavonoid biosynthesis, phenylalanine metabolism, photosynthesis, beta-alanine metabolism, fatty acid degradation, tyrosine metabolism, glycerolipid metabolism, carotenoid biosynthesis and thiamine metabolism (**Figure 8b**).

### Cluster analysis of DEGs

**Figure 9:**
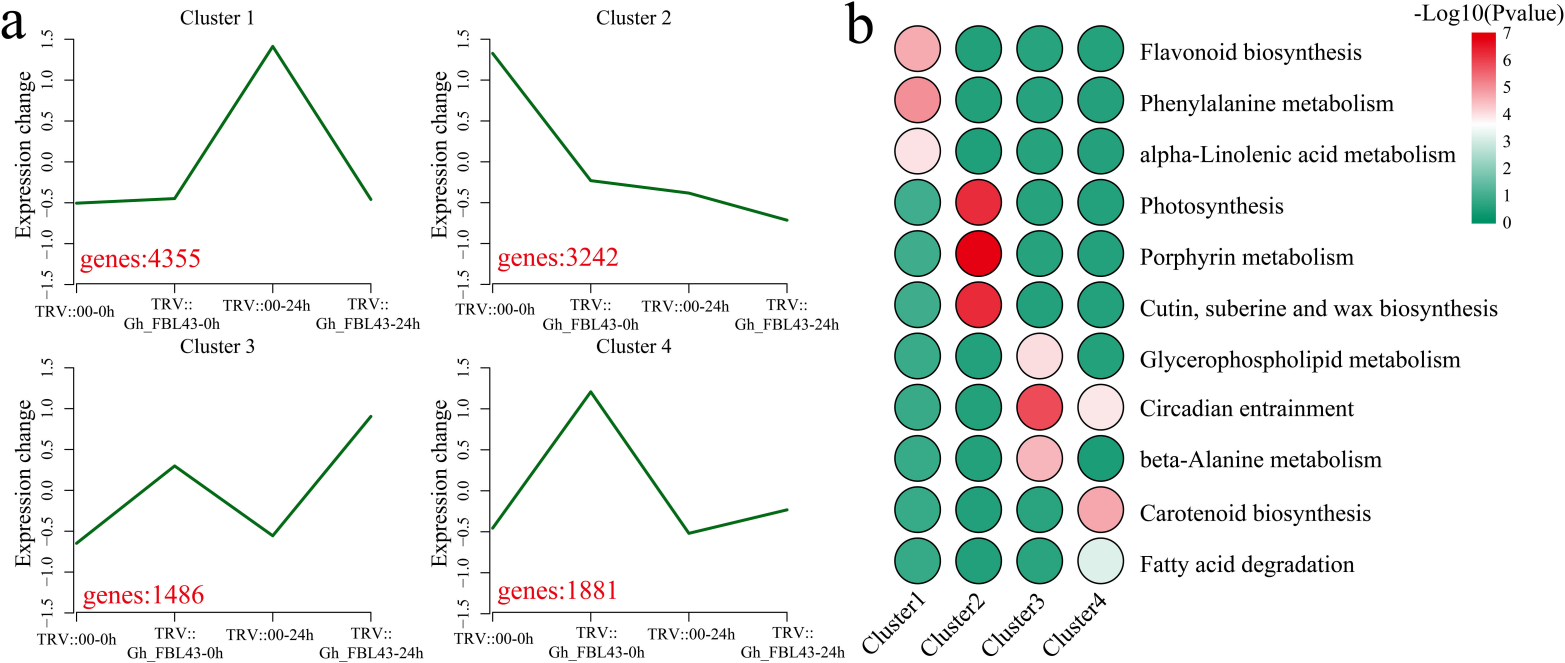
Clustering and KEGG enrichment analysis of DEGs between Gh_FBL43-silenced and control plants. **(a)** Clustering analysis of DEGs, with red numbers representing the number of DEGs contained in each cluster. **(b)** Heatmap of the results of the KEGG enrichment analysis for each cluster.

Using k-means clustering, the 10,928 DEGs were analyzed, and the pathways involved in each cluster were annotated. Four clusters with statistical significance were identified (**Figure 9a**). Cluster 1 presented the highest expression level at 24 hours post-inoculation in the control plants, encompassing 4,355 DEGs that were significantly enriched in the flavonoid biosynthesis, phenylalanine metabolism, and alpha-linolenic acid metabolism pathways (**Figures 9a, b**). Cluster 2 presented the highest expression level in the control plants prior to inoculation, with expression levels decreasing in both the control and silenced plants post-inoculation, with 3,242 DEGs that were significantly enriched in the photosynthesis, porphyrin metabolism, and cutin, suberin and wax biosynthesis pathways. Cluster 3 had the highest expression level at 24 h post-inoculation in the silenced plants, with a slight increase in expression levels following inoculation. Cluster 3 included 1,486 DEGs that were significantly enriched in the glycerophospholipid metabolism, circadian entrainment and beta-alanine metabolism pathways. Cluster 4 presented minimal changes in expression levels before and after inoculation in the control plants, however, the expression levels in the *Gh_FBL43*-silenced plants decreased post-inoculation. Cluster 4 included 1,881 DEGs that were significantly enriched in the circadian entrainment, carotenoid biosynthesis and fatty acid degradation pathways.

### Analysis of the biochemical synthesis pathways of JA and flavonoids

**Figure 10:**
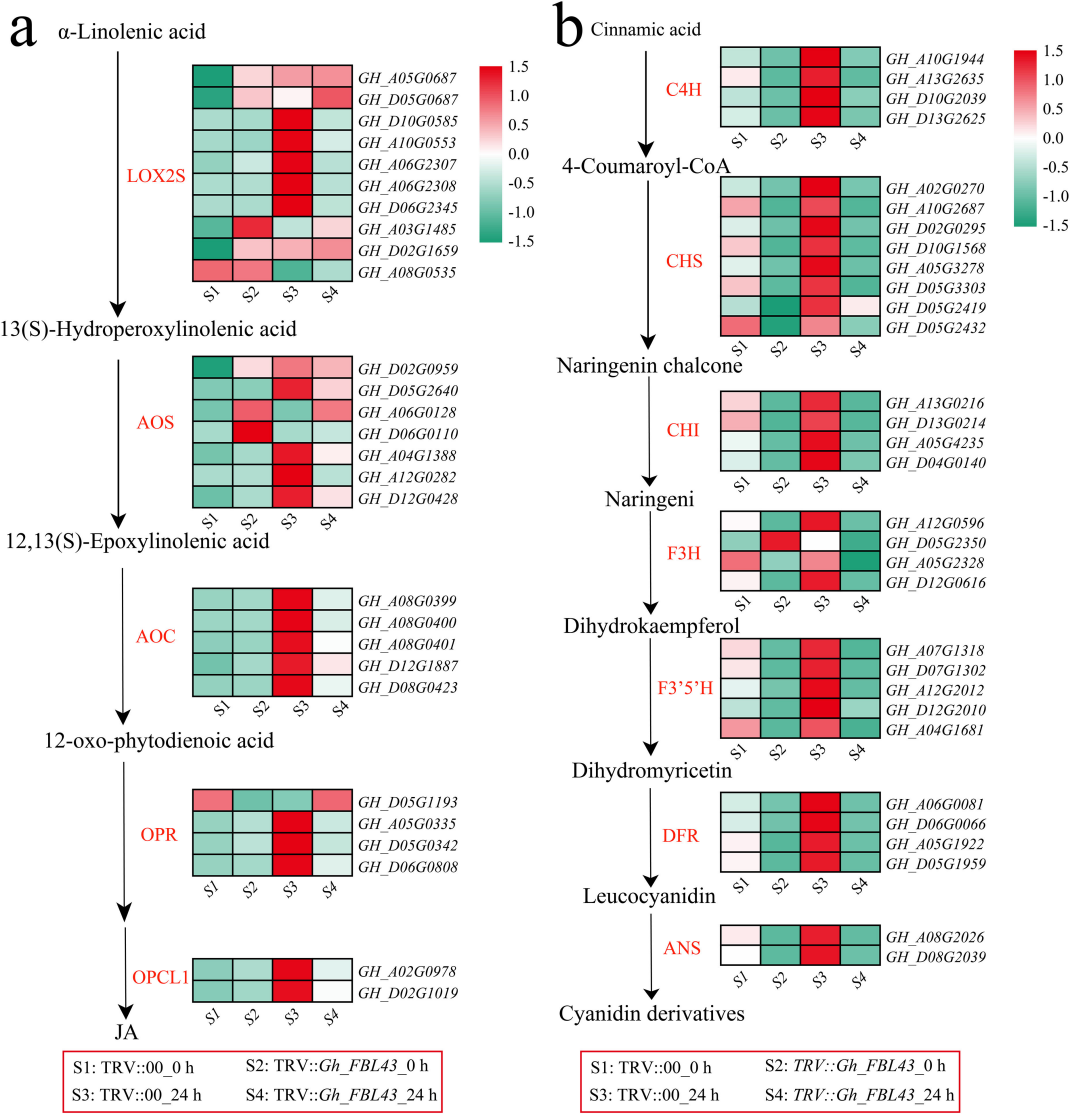
Analysis of the memory expression patterns of the JA and flavonoid pathways in the *Gh_FBL43*-silenced and control plants. **(a)** Analysis of gene expression patterns in the JA pathway, standardized scoring was used, with values standardized to range from -1.5 to 1.5. **(b)** Analysis of gene expression patterns in the flavonoid pathway, standardized scoring was used, with values standardized to range from -1.5 to 1.5.

The enrichment analysis of all the DEGs revealed that jasmonic acid (JA) metabolism, alpha-linolenic acid metabolism and flavonoid-related pathways were significantly enriched. The alpha-linolenic acid metabolism pathway is an important route for the synthesis of JA in plants. Therefore, the expression patterns of JA synthesis genes were first analyzed. The results indicated that the expression levels of most JA synthesis genes decreased after infection in the *Gh_FBL43-*silenced plants, whereas the control plants presented the highest expression levels 24 hours post infection (**Figure 10a**). Among the rate-limiting enzyme allene oxide synthase (AOS)-encoding genes involved in JA synthesis, all the genes except for *GH_A06G0128* and *GH_D06G0110* presented peak expression in the control plants at 24 hours post infection. Additionally, a gene encoding another rate-limiting enzyme, allene oxide cyclase (AOC), also presented peak expression in the control plants at this time point. With respect to the genes involved in the flavonoid synthesis pathway, all the genes except for *GH_D05G2350* (flavanone 3-hydroxylase, F3H) presented higher expression levels in the control plants than in the *Gh_FBL43*-silenced plants, with expression levels increasing with infection and peaking at 24 hours post infection in the control plants (**Figure 10b**). These results suggest that *Gh_FBL43* may regulate the resistance of *G. hirsutum* to wilt disease by modulating the expression of genes involved in JA and flavonoid biosynthesis.

## Discussion

In recent years, advancements in sequencing technology and the continuous reduction in sequencing costs have led to the ongoing refinement and updating of cotton genome sequencing efforts, laying a foundation for studying gene functions at the whole-genome level (Wang et al., 2019; Hu et al., 2019; Huang et al., 2020). Recently, the successful assembly and publication of the telomere-to-telomere (T2T) genomes of diploid *G. raymondii* and allotetraploid *G. hirsutum* (TM-1 and Zhongmian 113) have improved our understanding of cotton genomics and genetics, providing a solid foundation for exploring gene families in cotton and elucidating their phylogenetic relationships (Huang et al., 2024; Hu et al., 2025a, Hu et al., 2025b). Genetic evolutionary analyses indicate that *G. raymondii* and *G. arboreum* are the ancestors of the allotetraploid cotton species (*G. hirsutum* and *G. barbadense*) (Wang et al., 2019; Hu et al., 2019). FBL genes play crucial roles in plant growth and development, as well as in the response to various stresses, such as drought, salinity, and pathogens, and have been widely characterized in numerous plants (Elzanati et al., 2017). Therefore, the identification and characterization of the FBL gene family in *G. hirsutum*, *G. barbadense* and their two ancestral species will improve the understanding of the evolution and functions of FBL genes. This study identified FBL genes in cotton and revealed that the number of FBL family genes in *G. raymondii* is slightly greater than half that in *G. hirsutum*, possibly because of the use of the T2T genome of *G. raymondii*, which also indicates that the assembly of the T2T genome provides a basis for identifying additional new genes. An analysis of the number of FBL family genes and the results of the phylogenetic tree revealed that the FBL gene family has remained relatively conserved throughout the long evolutionary history of cotton.

The role of F-box-LRR genes in plant disease resistance has been well validated. The gene *FBS1*, which encodes an F-box protein in *A. thaliana*, positively regulates the resistance of *A. thaliana* to pathogens by activating the genes involved in JA biosynthesis (Gonzalez et al., 2017). Additionally, the F-box protein constitutive expression regulator (CPR1), which is associated with pathogenesis-related (PR) genes, modulates resistance to *Pseudomonas syringae* by inhibiting the accumulation of the R protein SNC1, acting as a negative regulatory factor in plant defense (Zribi et al., 2021). Furthermore, the rice F-box protein *OsFBX156* interacts with the heat shock protein *OsHSP71.1*, promoting its degradation, which activates the reactive oxygen species (ROS) and salicylic acid (SA) signaling pathways, thereby increasing rice disease resistance (Zhao et al., 2024). Although these studies indicate that F-box proteins play a significant role in plant defense against pathogens, the regulatory pathways involved differ. Expression pattern analysis revealed that the expression level of *Gh_FBL43* significantly increased following the occurrence of Verticillium wilt, and the difference in expression level between resistant and susceptible materials was statistically significant. Silencing of *Gh_FBL43* in plants resulted in decreased resistance to Verticillium wilt, indicating that *Gh_FBL43* is a positive regulatory gene for Verticillium wilt resistance in *G. hirsutum*.

Flavonoid synthesis occurs through the phenylpropanoid pathway and involves the collaborative action of related genes such as those encoding chalcone isomerase (CHI) and chalcone synthase (CHS) (Liu et al., 2021). Within plants, flavonoid metabolites can exhibit antioxidant effects, including disease and pest resistance, ultraviolet resistance, and free radical scavenging effects (Ramaroson et al., 2022). Additionally, flavonoids can modulate the immune signaling pathways of plants, activating defense mechanisms that facilitate a rapid response to pathogenic infections, thus increasing disease resistance (Kumar et al., 2023). Treatment with exogenous kaempferol and apigenin at the spike significantly increased wheat resistance to fusarium head blight caused by *Fusarium graminearum* (Zhao et al., 2022). Research has shown that anthocyanins, such as glycosylated cyanin 3-D-galactoside and 7-O-methyl-cyanin 3-O-β-D-pyranagalactoside, as well as flavonols, are present at relatively high concentrations on the red side of red mango fruit, with the red side proving more effective than the green side in inhibiting Colletotrichum (Sudheeran et al., 2021). Glucosidase can hydrolyze anthocyanins and flavonols, with the generated glycoside moieties exhibiting strong toxicity against fungal pathogens (Sudheeran et al., 2020). In the *PalbHLH1* and *PalMYB90* overexpression lines of poplar, structural genes related to flavonoids, such as F3H, FLS, DFR, ANS, and ANR, were upregulated, promoting the accumulation of quercetin, kaempferol, and anthocyanins and thereby increasing the resistance of poplar trees to *Melampsora larici-populina* (Bai et al., 2020). In soybean, knocking out *GmF3H1*, *GmF3H2* and *GmFNSII-1* can increase the isoflavone content, thus improving the resistance of soybean leaves to soybean mosaic virus (Zhang et al., 2020). Enrichment analysis of the DEGs revealed significant enrichment in the biosynthetic pathways of flavonoids and phenylpropanoids, with genes in the flavonoid synthesis pathway showing higher expression levels in the control plants than in the *Gh_FBL43-*silenced plants, and their expression increased under pathogen inoculation. These results suggest that *Gh_FBL43* may regulate the resistance of *G. hirsutum* to yellow wilt by modulating the expression of flavonoid biosynthesis genes, indicating the crucial role of flavonoids in the resistance of *G. hirsutum* to yellow wilt.

Hormones, as signaling molecules within plants, play crucial roles in signal transduction processes under pathogen stress (Verma et al., 2016). Among these, JA plays a critical role in regulating plant disease resistance-related signaling pathways (Ding et al., 2022). The synthesis of JA is initiated by the release of α-linolenic acid from membrane lipids through various enzymes involved in the lipoxygenase pathway (Huan et al., 2022). Research conducted in rice has shown that viral transcription suppression factors (*RBSDVP8, SRBSDVSP8, RSVP2* and *RSMVM*) inhibit the key functional module *OsJAZ*-*OsMYC3*-*OsMED25* in the JA signaling pathway, thereby decreasing disease resistance in rice (Li et al., 2021). *OsGSK2* inhibits the repressor *OsJAZ4* through phosphorylation and promotes its degradation, thereby activating the JA response and the JA mediated antiviral defense response in rice and increasing the resistance to black-streaked dwarf disease (He et al., 2020). *A. thaliana* mutants deficient in JA synthesis (*fad3/fad7/fad8*, *dde1/opr3* and *dde2/aos*) exhibit significantly weakened resistance to pathogenic fungi (Chini et al., 2007; McConn and Browse, 1996; von Malek et al., 2002). Furthermore, JA can be transported over long distances within vascular bundles and via air, transmitting invasion signals from infected sites to the entire plant and thereby inducing systemic resistance (Li et al., 2020). Analysis of DEGs before and after inoculation of the control plants and *Gh_FBL43*-silenced plants revealed significant enrichment in the α-linolenic acid and JA metabolic pathways. The expression of the rate-limiting enzymes for JA synthesis, AOC (G*H_A08G0399, GH_A08G0400, GH_A08G0401, GH_D12G1887* and *GH_D08G0423*) and AOS (*GH_D02G0959, GH_D05G2640, GH_A04G1388, GH_A12G0282* and *GH_D12G0428*), was highest in the control plants 24 hours post-inoculation. In conclusion, these comprehensive results suggest that *Gh_FBL43* may regulate the expression of JA and flavonoid biosynthesis genes to modulate resistance to wilt disease in *G. hirsutum*.

## Conclusion

By studying the origin and evolution of genes and performing functional analysis, sufficient information can be gained for further mechanistic elucidation. This study identified 57 FBL genes in the *G. hirsutum* genome. These genes can be classified into five subgroups and were relatively conserved during the evolution of cotton. Expression analyses combined with VIGS revealed that *Gh_FBL43* is associated with resistance to Verticillium wilt in *G. hirsutum*. RNA-seq analysis revealed that silencing of *Gh_FBL43* reduced the expression of genes involved in JA and flavonoid-related pathways. In summary, our findings demonstrate that the *Gh_FBL43* gene plays a significant role in the resistance of *G. hirsutum* to Verticillium wilt and that JA- and flavonoid-related pathways are important contributors to this resistance, identifying new genes and potential regulatory pathways for further research on *G. hirsutum* resistance to Verticillium wilt.

## Data Availability

The RNA-seq data used in the study have been uploaded to the Genome Sequence Archive of the National Genomics Data Center, China National Center for Bioinformation, under project PRJCA039530.

## Supplementary material

Supplementary Figure 1 Chromosomal localization of FBL genes in *G. hirsutum.*
