# Supplementary figures and images for: Gh_FBL43 regulates the resistance of Gossypium hirsutum to Verticillium wilt through jasmonic acid and flavonoid-related pathways
Source: Front Plant Sci. 2025 Jul 17;16:1620947. doi: 10.3389/fpls.2025.1620947 (PMC12310711; doi:10.3389/fpls.2025.1620947)

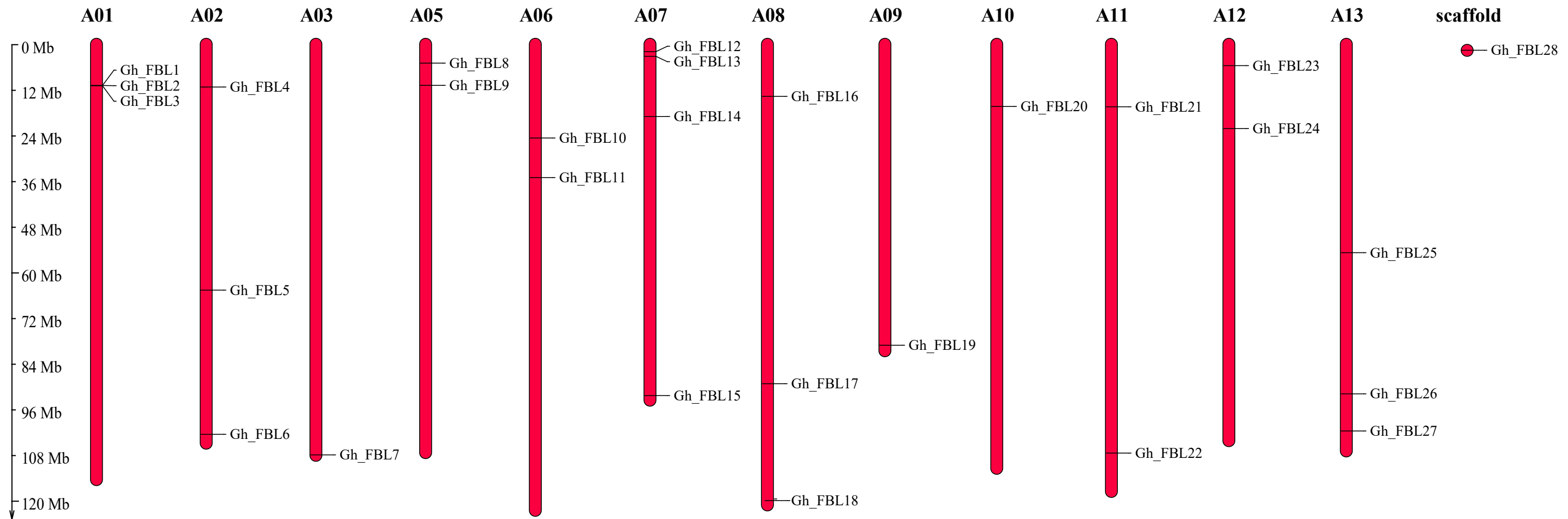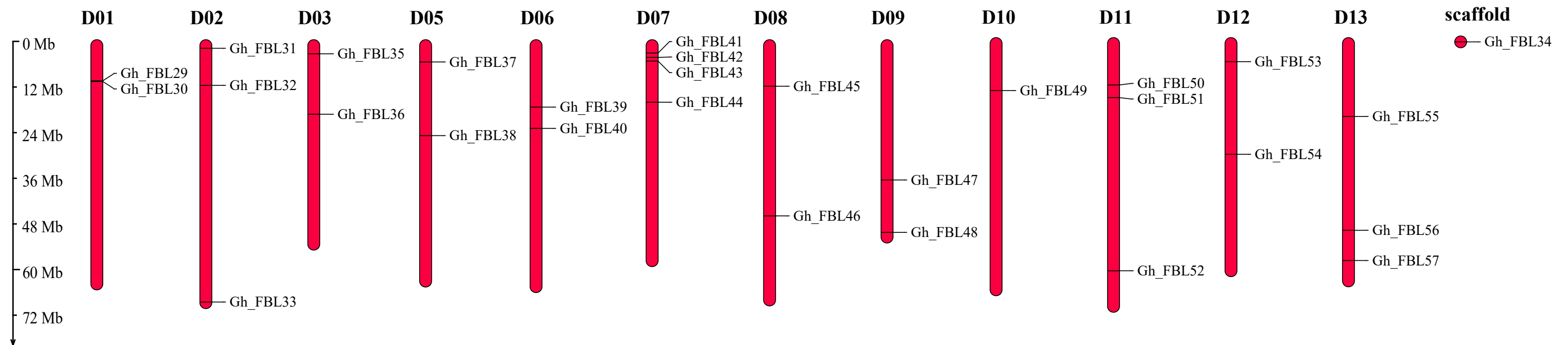

Supplement: Supplementary Figure 1 — Chromosomal localization of FBL genes in G. hirsutum. [file DataSheet1.pdf]
